# ConcatSeq: A method for increasing throughput of single molecule sequencing by concatenating short DNA fragments

**DOI:** 10.1038/s41598-017-05503-w

**Published:** 2017-07-12

**Authors:** Ulrich Schlecht, Janine Mok, Carolina Dallett, Jan Berka

**Affiliations:** Roche Sequencing Solutions, 4300 Hacienda Drive, Pleasanton, CA 94588 USA

## Abstract

Single molecule sequencing (SMS) platforms enable base sequences to be read directly from individual strands of DNA in real-time. Though capable of long read lengths, SMS platforms currently suffer from low throughput compared to competing short-read sequencing technologies. Here, we present a novel strategy for sequencing library preparation, dubbed ConcatSeq, which increases the throughput of SMS platforms by generating long concatenated templates from pools of short DNA molecules. We demonstrate adaptation of this technique to two target enrichment workflows, commonly used for oncology applications, and feasibility using PacBio single molecule real-time (SMRT) technology. Our approach is capable of increasing the sequencing throughput of the PacBio RSII platform by more than five-fold, while maintaining the ability to correctly call allele frequencies of known single nucleotide variants. ConcatSeq provides a versatile new sample preparation tool for long-read sequencing technologies.

## Introduction

The cost for sequencing DNA has decreased dramatically over the course of the last ten years at a rate outpacing Moore’s law. While we are fast approaching an era in which sequencing an entire human genome costs less than $1,000^1^, it currently still is not feasible to decipher large numbers of complex genomes due to reagent costs, informatics infrastructure, time for sample preparation, and sequencing. To this end, multiple ‘target enrichment’ methods have been developed in recent years to selectively enrich for parts of the genome that contain relevant information of interest^2^. These enrichment methods offer effective ways to lower sequencing cost, increase sequencing depths, shorten sequencing time, and simplify data analysis, and thus have been routinely applied to study human disease. Among the most popular enrichment methods are multiplex PCR^3^, molecular inversion probes^4–7^, and hybrid capture^8–12^. Typically, target enrichment generates sequencing libraries that contain short DNA molecules (100–300 bp) ideally suited for short-read sequencing platforms such as Illumina’s benchtop sequencer suite. However, alternative long-read sequencing platforms such as single molecule sequencing (SMS) technologies developed by Pacific Biosciences^13, 14^ (PacBio) or Oxford Nanopore Technologies^15–17^ (ONT), are gaining traction because of their ability to more easily detect complex structural variations, and characterize extended repetitive regions in the genome^18^.

PacBio’s single molecule real-time (SMRT) technology distinguishes itself from other sequencing platforms in three main aspects: (1) during library preparation, closed circular DNA molecules are created by ligating hairpin adapters, termed SMRTbells, to both ends of double-stranded DNA target molecules, (2) these SMRTbells are bound to a sequencing primer and a DNA polymerase, and subsequently loaded as a complex into tiny sequencing units called zero-mode waveguides (ZMWs), and (3) the small volumes of the ZMWs allow real-time optical detection of fluorescently-labeled phospholinked nucleotides as they are incorporated by the DNA polymerase while a copy of the template is synthesized^18^. Because of the circular nature of the SMRTbell-ligated library and the DNA polymerase’s capacity to generate reads longer than multiple kilobases, sequencing is not limited to one template strand but instead extends to multiple passes across both strands. The information from these multiple passes mitigates the relatively high error rate per single pass and is used to generate a circular consensus sequence (CCS) read with high accuracy.

One limitation of long-read sequencing technologies is currently their low throughput. For example, on the commercially available PacBio RSII system the number of reads generated per run are typically in the tens of thousands^18^. A new generation of the platform (the Sequel System) is projected to increase the sequencing throughput by approximately seven-fold^19^, but will still be at least ten times lower in throughput compared to short-read sequencers. This presents a challenge to PacBio and other SMS platforms when considering sequencing applications in which a large number of short DNA molecules, such as circulating tumor DNA^20^ or DNA extracted from formalin-fixed paraffin-embedded tissues^21^, need to be sequenced. One solution to address this challenge, however, is to concatenate short DNA fragments into long DNA templates. Such an approach would not only increase the throughput of single molecule sequencers with short DNA molecules, but also increase their versatility in being able to sequence both long and short DNA molecules in a cost-effective way.

In recent years, the synthetic biology community has developed various molecular biology methods to concatenate DNA fragments into genes or gene clusters for the purpose of genome engineering and the production of high added value biomolecules such as pharmaceuticals and biofuels^22–24^. For example, Golden Gate Assembly uses Type IIS restriction enzymes to cleave DNA proximal to their recognition site and enables the directed assembly of fragments with the correct orientation^25^. Cycled Ligation Assembly is an alternative approach that employs a thermostable DNA ligase and bridging oligonucleotides that have homology to the beginning and end of two adjacent DNA fragments^26^. However, the most widely adopted method is Gibson Assembly^27, 28^ due to its ease of use and versatility. In Gibson Assembly, three enzymes, a 5’ exonuclease, a DNA polymerase, and a DNA ligase, are used to covalently link DNA fragments with complementary ends in a simple one-pot isothermal reaction. In most Gibson Assembly applications, the concatenated fragments are cloned into a vector and subsequently passaged through bacteria for sequence-verification of the desired construct. The method has been demonstrated to work robustly for seamless concatenation of up to 6 fragments that can range in size from 100 bp up to hundreds of kilobases, while introducing minimal errors during *in vitro* recombination^27^.

Here, we present a novel method for SMS library preparation that concatenates pools of short DNA fragments (ranging between ~80 and 800 bp in size) into long concatemers using Gibson Assembly and demonstrate that our approach is capable of increasing the sequencing throughput of the PacBio RSII platform by more than five-fold. We validated our approach using a multiplex PCR panel for known single nucleotide variants in major oncology targets and show that allele frequencies are correctly called. In addition, we show an adaptation of this method to a hybrid capture-based target enrichment workflow. The method described here offers a novel approach to expand the versatility of SMS platforms by increasing the sequencing throughput of short fragments on these otherwise long-read sequencing technologies.

## Results

### Gibson Assembly concatenates short DNA amplicons into long concatemers

**Figure 1:**
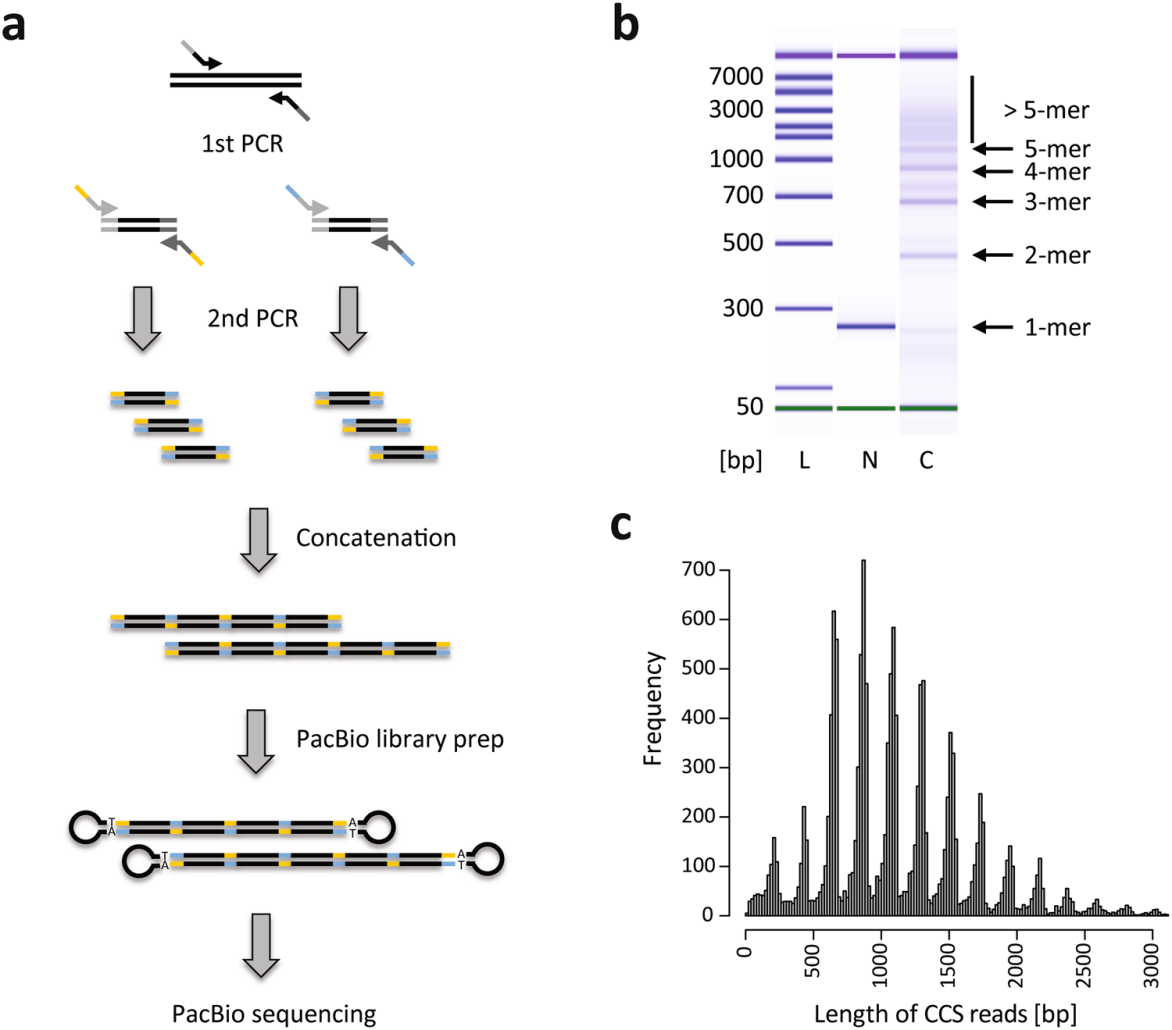
Gibson Assembly concatenates short DNA amplicons into long concatemers. (**a**) Schematic of the ConcatSeq sample preparation workflow for PacBio sequencing. A target amplicon is generated by PCR using primers flanked with spacer sequences (gray portions). These products are then used for a second round of PCR, where in two separate reactions, two different primer sets are used to incorporate complementary Gibson Assembly-compatible adapters to the target molecules (blue and yellow portions). Portions depicted in blue are the reverse complement of those depicted in yellow. The two amplicon pools are then mixed at equimolar quantities and incubated with an enzyme master mix that carries out the Gibson Assembly to generate a pool of concatemers of various lengths and randomized composition. Concatenation is followed by library preparation that attaches PacBio-specific hairpin adapters via A-tailed ligation and subsequent sequencing on a PacBio RSII instrument. (**b**) Bioanalyzer DNA7500 gel image showing ladder [L], the non-concatenated amplicon pool [N], and the concatenated sample [C]. Banding pattern in the concatenated sample indicates depletion of monomers and accumulation of n-mers of higher degree. The expected size of n-mers created this way corresponds to (n * x) − ((n−1) * y) where x is the size of a monomer and y is the size of the adapter. As in the example shown here the size of a monomer is 247 bp, the expected sizes are: 464, 681, 898, 1115, 1332, and 1549 bp for 2- to 7-mers. (**c**) Histogram of the length of PacBio circular consensus sequences (CCS reads) of the concatenated sample. Peaks coincide with the expected sizes of n-mers. For clarity, the histogram was truncated at 3 kb.

Covalent linkage of DNA molecules via Gibson Assembly is based on the existence of overlapping adapter sequences at their ends. We designed a 30 bp long adapter sequence with a GC-content of exactly 40%, which previously had been reported to be optimal for Gibson Assembly reactions^29^. As a proof-of-principle, we used a portion of exon 3 from the human NRAS locus spanning 120 bp to serve as a model target sequence. Target DNA molecules flanked with our overlapping adapter sequence were amplified using two rounds of PCR (Supplementary Table S1 lists all primers used in this study). The first round PCR incorporated spacer sequences up- and downstream of the amplicon and resulted in a product 187 bp in size (Fig. 1a). The second round PCR added two types of adapters in two separate reactions, resulting in a final product size of 247 bp (Fig. 1b, lane [N]). These adapter-flanked amplicons were then mixed at equimolar concentrations and incubated with a commercial Gibson Assembly master mix^30^ for 1 hour. In contrast to traditional uses of Gibson Assembly where fragments are assembled in a specified order, concatenation in our approach occurs in a random fashion (Supplementary Fig. 1); new units can be added to both ends of a growing concatemer chain. Monomers are increasingly depleted, and concatemers of higher degrees (such as dimers, trimers, tetramers et cetera, collectively termed n-mers) are generated (Fig. 1b, lane [C]). In fact, the observed lengths of the n-mers are nearly exactly of the expected sizes.

Next, we used the KAPA Hyper Prep library preparation kit to prepare a sequencing library by attaching PacBio-compatible hairpin adapters (SMRTbells) to the ends of the concatemers via A-tailed ligation (Fig. 1a). The library was sequenced on a PacBio RSII instrument. Subreads were summarized into Circular Consensus Sequence (CCS) reads using the standard Read Of Insert (ROI) algorithm on the SMRT Portal website. This approach identified 14,739 independent CCS reads (referred to as ‘reads’ from here on). As expected, the length distribution of the reads closely followed the size distribution of the underlying n-mers (Fig. 1c). Supplementary Table S2 gives an overview of all steps involved in creating sequencing libraries using the ConcatSeq method.

### ConcatSeq can increase sequencing throughput by more than five-fold

**Figure 2:**
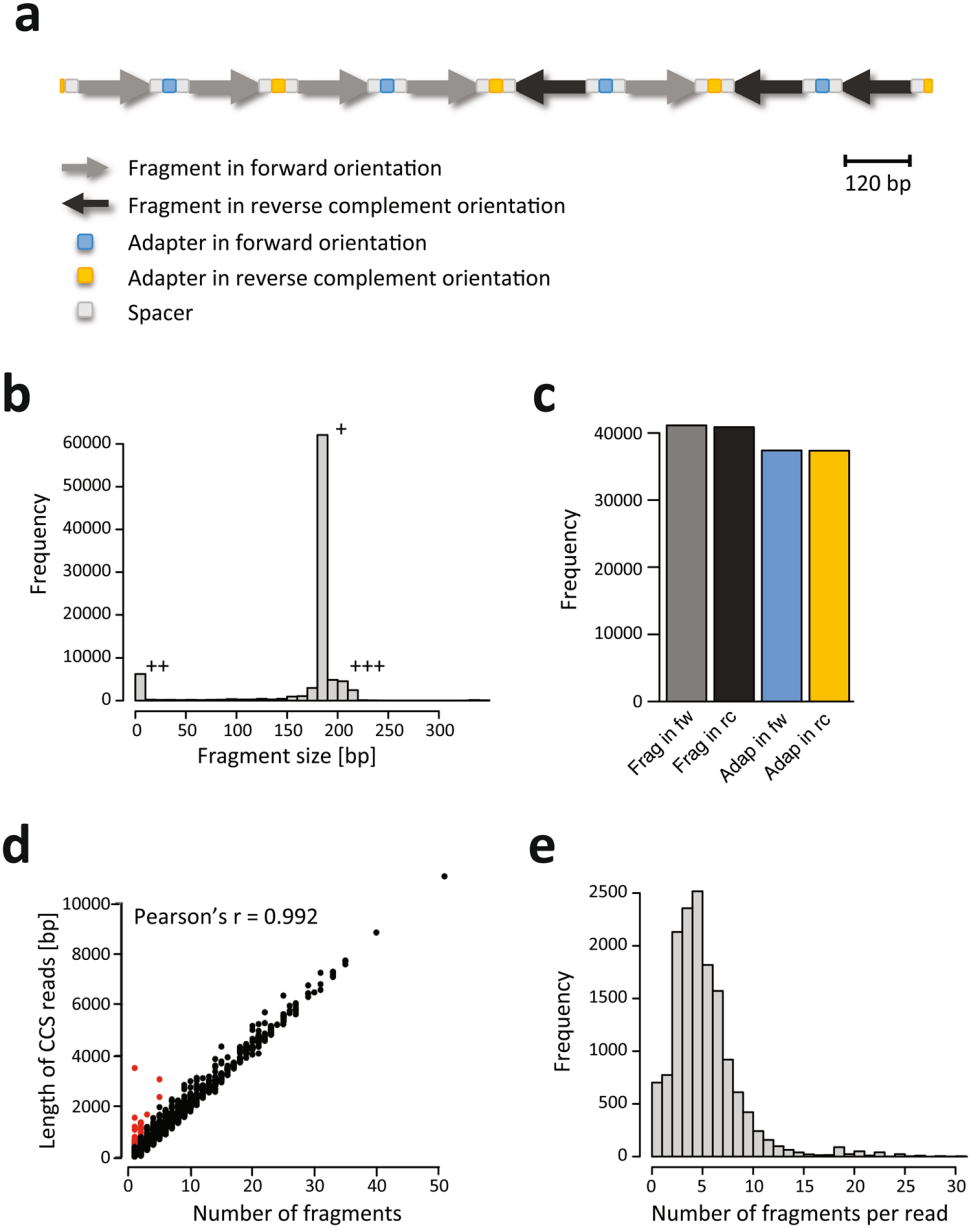
ConcatSeq can increase sequencing throughput by more than five-fold. (**a**) Schematic of the architecture of one example read (read number 5 in SRR5168830), depicting types and orientation of different sequence features identified: fragments and adapters in forward and reverse complement orientation, and spacers. (**b**) Histogram depicting the frequency of fragments in each size bin (10 bp interval) after deconcatenation of sequencing reads using the adapter scanning approach. +: fragments with exactly (187 bp) or very close to the expected size (180–190 bp); ++: fragments that are shorter than 10 bp; +++: fragments that are slightly larger than the expected size (>190 bp). (**c**) Barplot depicting the number of fragments [Frag] that aligned to the reference and adapters [Adap] that were identified by the adapter scanning approach in forward [fw] and reverse complement [rc] orientation in all reads. (**d**) Scatterplot depicting the relationship between read length and number of fragments identified in that read. Red dots indicate the cases in which the read was significantly longer than the length expected by the number of fragments identified in that read. (**e**) Histogram depicting the frequency of number of fragments identified per read across all reads. For clarity, the histogram was truncated at 30 fragments per read.

To confirm that our concatenation approach was successful, we randomly chose a read consisting of 1,719 bp (read number 5 in SRR5168830) for detailed inspection. Based on its length, we suspected and confirmed this read to be an 8-mer. From the design used in our approach, we furthermore expected recurring features to be identified in this read: the 30 bp ConcatSeq adapters, target sequence, and spacers (Fig. 2a; for simplicity, the target plus flanking spacer sequences will be referred to as ‘targets’ or ‘fragments’ from here on). We also expected adapters switch between forward and reverse complement orientation along the read. All three recurring features and adapter switching between forward and reverse complement orientation were observed. Because the target units can be concatenated in random orientation, we observed targets present in both orientations but at roughly the same frequency (*i.e*. five in forward and three in reverse complement).

To extend this type of analysis to all 14,739 sequencing reads, we implemented a bioinformatics method to automate deconcatenation. This method is based on an algorithm where a scanning window slides along each read and makes an approximate matching (with up to 4 mismatches tolerated including deletions and insertions) to the expected adapter sequence, then generates lists of adapter and fragment positions in every read (Supplementary Data S1 contains the R-script used for deconcatenation). In sum, 89,496 fragments and 75,312 adapters were identified in the 14,739 reads. The vast majority of the fragments (n = 62,093, 74.2%) had exactly the expected size of 187 bp or was very close (181–190 bp) to the expected size (Fig. 2b). Notably, there was a second population of fragments that consisted of only one base (n = 5,818, 6.5%). All of these fragments were located at the beginning or the end of the reads, and the majority of these (85%) were either an adenine or a thymidine. These single base fragments are most likely remnants of the hairpin adapters attached to the n-mers via A-tailed ligation during library preparation (Fig. 1a). A third population (n = 12,783, 15.3%) consisted of fragments that were slightly longer than the expected size (>190 bp). Again, the majority of these fragments was located at the ends of the reads and contained the target along with truncated adapter sequences.

We excluded the 5,818 single-nucleotide fragments from further analysis, leaving 83,678 fragments after deconcatenation (Table 1). On average, each read contained 5.68 fragments, indicating that our approach increased the sequencing throughput by more than five-fold compared to sequencing a pool of non-concatenated fragments. Alignment of the targets to the reference sequence showed a very high on-target rate (98.0%; *i.e*. 82,008 of the 83,678 fragments), suggesting that concatenation did not interfere with the fidelity of the target sequences. As expected, the number of all aligned fragments in forward and reverse complement orientation was almost exactly equal (41,132 and 40,876, respectively; Fig. 2c). The same was true for the adapters in both orientations. We observed a smaller number of adapters compared to fragments, which we hypothesized was due to truncated adapters that are located at the ends of the concatemers. Such cases would not be identified by our adapter scanning approach. Further inspection of the ends of the reads confirmed this hypothesis.

**Table 1:** Overview of PacBio sequencing runs.

Figure(s)	DNA source	# of reads	# of fragments^a^	degree of concatenation^b^	# of aligned fragments	on-target rate^c^	SRA Acc^d^
1 and 2	NRAS (exon 3)	14,739	83,678	5.68	82,008	98.0%	SRR5168830
3	Cancer panel (NC)	15,143	15,143	1	14,700	97.1%	SRR5168831
3	Cancer panel (C-1)	18,561	98,250	5.29	94,892	96.6%	SRR5168832
3	Cancer panel (C-2)	26,601	134,146	5.04	128,971	96.1%	SRR5168833
3	Cancer panel (C-3)	20,686	108,078	5.22	104,562	96.7%	SRR5168834
4	EGFR locus	52,341	231,801	4.43	224,595	96.9%	SRR5168838
Supp. Figure 2	LMW DNA ladder	48,183	181,901	3.78	148,300	81.5%	SRR5168841

‘# of’ stands for ‘number of’;

NC: non-concatenated pool; C-1,2,3: concatenated pool, replicates 1,2,3;

^a^this excludes all fragments that are only 1 bp long;

^b^this is the ratio of # of fragments and the # of total reads;

^c^this is the ratio of # of aligned reads and the # of fragments;

^d^this is the accession number for sequence data deposited in the SRA (Sequence Read Archive).

Because the fragments that were concatenated in this experiment were all of the same size (Fig. 1b, lane [N]), there should be a linear relationship between the length of the read and the number of fragments identified in that read. This linear relationship was observed for the large majority of reads (Fig. 2d). In the remaining 22 reads (red data points in Fig. 2d), a few adapter sequences failed to be identified by our algorithm because they had more than 4 mismatches with the reference sequence. Strikingly, while the majority of reads (70.5%) contained between three and seven fragments (Fig. 2e) and were between 600 and 1,500 bp in length, we found a wide spread of read lengths, with the longest being more than 10 kb in size and containing more than 50 fragments (Fig. 2d). This suggests that if size selection for longer concatemers prior to sequencing is performed, ConcatSeq has the potential to further increase the sequencing throughput.

We would like to note that a small number of fragments (409 to be exact, which corresponds to ~0.49% of all fragments) contained unexpected structures. These appeared to be hybrid sequences of two or more fragments that were concatenated outside of the Gibson Assembly adapter region (see Methods). The occurrence of such hybrids could potentially be explained by excessive exonuclease activity that opens up and links regions of homology within the fragment sequences. For future improvements of ConcatSeq we envision strategies that prevent chewing of the exonuclease into the fragments (for example by incorporating phosphorothioate bonds between the adapter and the insert sequences) and that therefore suppress generating these undesired concatemers.

### ConcatSeq correctly identifies single-nucleotide variants (SNVs) in an oncology amplicon panel

**Figure 3:**
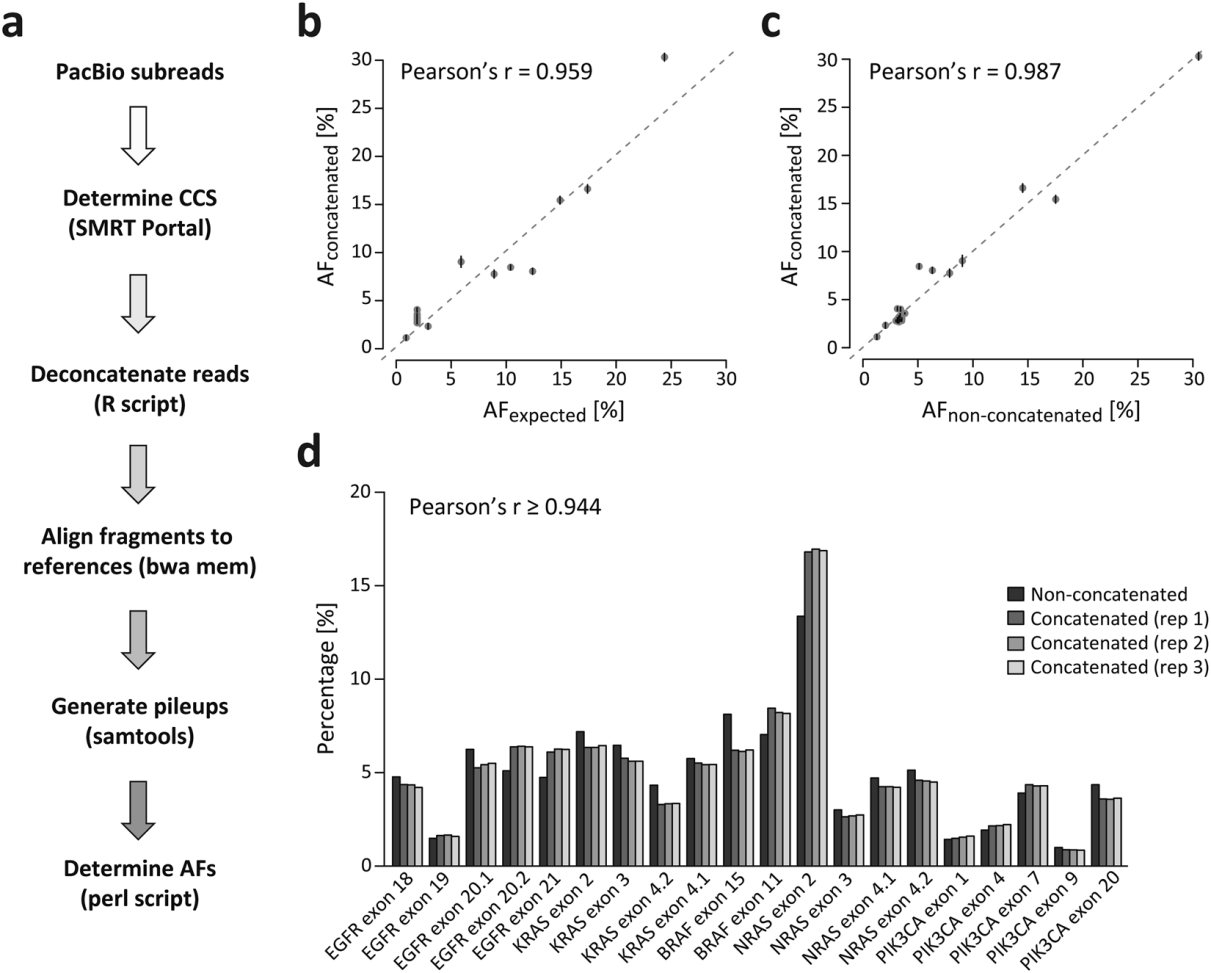
ConcatSeq correctly identifies single-nucleotide variants (SNVs) in an oncology amplicon panel. (**a**) Schematic depicting the bioinformatics analysis pipeline describing all steps and tools used, starting with PacBio raw reads (subreads) to determining allele frequencies (AFs) of known SNVs in HD701. (**b**) Scatterplot showing comparison of AFs identified in replicates of concatenation samples plotted against the expected frequencies. Average values from three independent experiments are shown. Error bars indicate standard deviation from the three measurements. (**c**) Scatterplot showing a comparison of AFs identified in replicates of concatenation samples plotted against frequencies found in the non-concatenation control sample. Average values from three independent experiments are shown. Error bars indicate standard deviation from the three measurements. (**d**) Barplot comparing amplicon coverage in non-concatenated and three replicates of concatenation samples. Frequencies were calculated by dividing the number of fragments that aligned to each of the 20 amplicons by the total number of aligned fragments. Pearson’s r was calculated for every replicate independently, and the lowest of the three correlation coefficients is indicated in the plot.

We next examined whether ConcatSeq can be used to correctly identify known SNVs and their allele frequencies in a biological sample. To this end, we amplified a set of oncology targets by PCR using a well-characterized DNA reference (HD701^31^, Horizon Discovery) as the template. HD701 is a commercially available engineered cell line in which precise allelic frequencies for major oncology targets have been determined by digital PCR. Allele frequencies (AFs) of the verified variants in this DNA sample are between 1% and 24.5%, allowing the assessment of the sensitivity of our assay. Twenty amplicons spanning 5 genes (EGFR, KRAS, NRAS, BRAF, and PIK3CA) were generated in two separate multiplex PCRs (containing 11 and 9 different targets, respectively), and then flanked by complementary ConcatSeq adapters by a second round of PCR. Equimolar amounts of these two amplicon pools were mixed and concatenated in three independent reactions, followed by PacBio RSII sequencing, serving as triplicate samples to assess reproducibility of our assay. As before, on average more than 5 fragments were observed per read in these samples (Table 1). We also sequenced the non-concatenated amplicon sample as a control. A bioinformatics pipeline was then established (Fig. 3a) that aligns the deconcatenated and non-concatenated fragments to the 20 reference sequences (Supplementary Table S3), generates pileups of each alignment, and subsequently extracts AFs of the known variants in HD701 cell line DNA. The on-target rates in all three concatenated samples and the non-concatenated control were again very high (≥96.1%). Allele frequencies identified with ConcatSeq were highly correlated (Pearson’s r = 0.959) between the three replicates of concatenated samples and the expected frequencies (Fig. 3b and Supplementary Table S4), indicating ConcatSeq’s ability to correctly retrieve this information with great sensitivity. A comparison of AFs in the concatenated samples and the non-concatenated control (Fig. 3c) showed even higher concordance (Pearson’s r = 0.987), indicating that deviations from the expected frequencies were likely introduced during amplicon generation and not during concatenation or PacBio sequencing. To ensure that our approach does not introduce a large bias into the frequency of amplicons represented in the pool before concatenation, we compared percent coverage of each of the 20 amplicons in the three concatenated samples and the non-concatenated control (Fig. 3d). We found a high correlation (Pearson’s r ≥ 0.944) between these groups, indicating that ConcatSeq does not introduce a large bias during subsampling from the original fragment pool. However, we noticed some level of variation: for 15 of the 20 amplicons the frequencies observed in the concatenated samples deviated at most 20% from those observed in the non-concatenated control. For the remaining 5 amplicons the observed difference was at most 32%.

### Adaptation of the current approach to other target enrichment workflows

**Figure 4:**
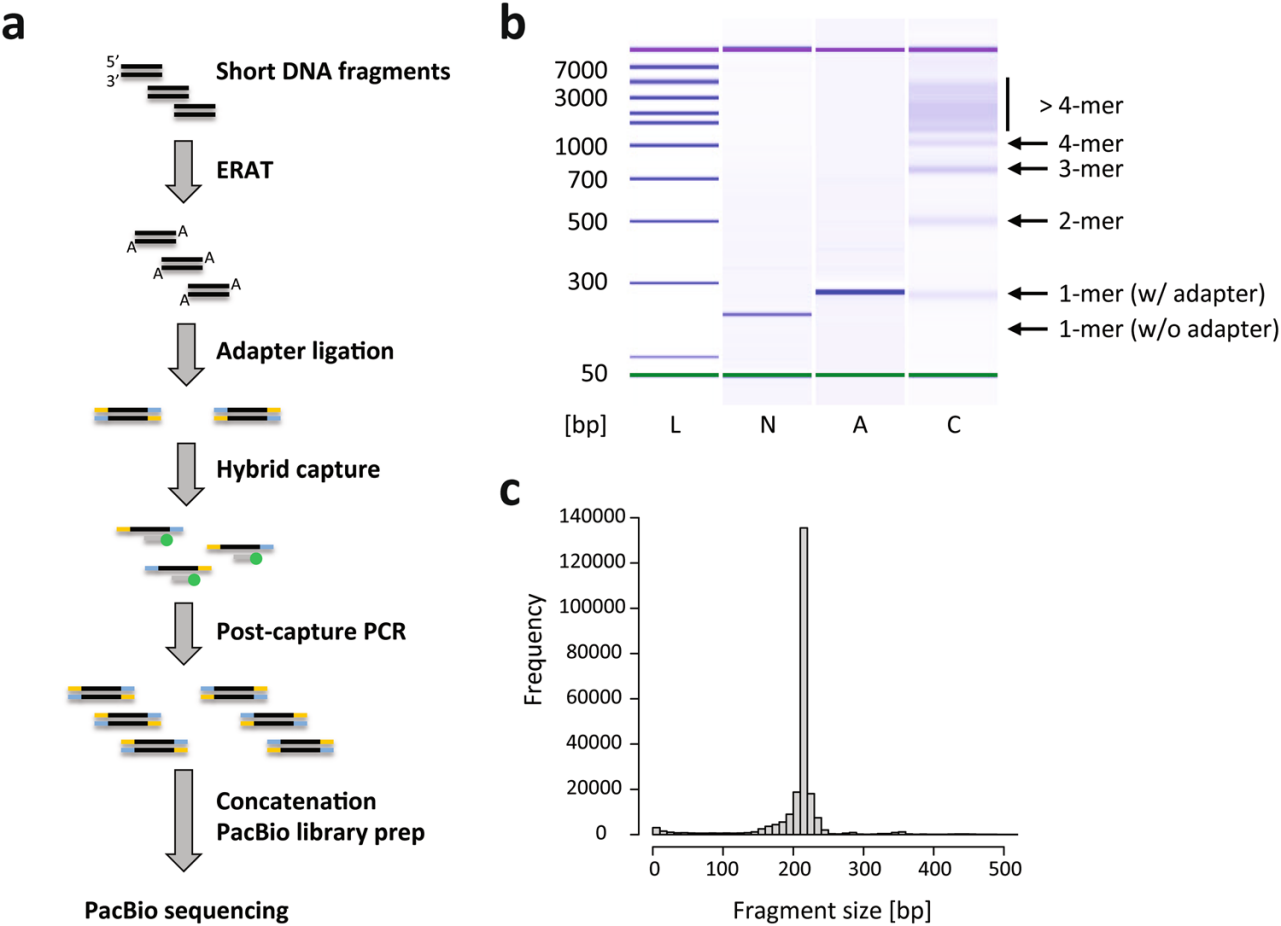
Adaptation of Concat-Seq to an alternative target enrichment workflow. (**a**) Schematic of Roche Nimblegen’s SeqCap workflow and its adaptation to ConcatSeq. Short DNA fragments, for example cell-free DNA, are prepared for adapter ligation by end-repair and A-tailing (ERAT). During adapter ligation, two types of ConcatSeq adapters are used (blue and yellow portions) instead of the Y-shaped adapters normally used in this step. The resulting library pool is then used for hybridization to biotinylated (green dots) hybrid capture probes (gray bars), and enriched targets are subsequently amplified in a post-capture PCR. The amplified material is then concatenated and processed as described in Fig. 1a. (**b**) Bioanalyzer DNA7500 gel image showing ladder [L], the non-concatenated amplicon pool (a pool of 4 amplicons from the EGFR locus) [N], the non-concatenated amplicon pool with adapters [A], and the concatenated sample [C]. As expected a shift of about 60 bp was observed between lanes [N] and [A] indicating successful ligation of ConcatSeq adapters to the original pool of amplicons. Banding pattern in lane [C] indicates depletion of monomers and accumulation of n-mers of higher degree. (**c**) Histogram depicting frequencies of fragment lengths after deconcatenation of EGFR-concatemer reads. The size of the vast majority of fragments coincides with the expected EGFR amplicon length (220 bp).

Multiplex PCR amplification is just one method of target enrichment for next-generation sequencing. Other strategies exist, including hybrid capture (such as Roche Nimblegen’s SeqCap^32^) and molecular inversion probe technologies (such as Roche Nimblegen’s HEAT-Seq^33^). We thus explored whether ConcatSeq could be adapted to these other methods while minimally modifying their workflows. Figure 4a depicts an adaptation for SeqCap in which there are only two changes to the standard workflow. First, the Y-shaped adapters, which are ligated to DNA fragments at the beginning of the protocol, are replaced with ConcatSeq adapters (Fig. 4a, Adapter Ligation step). Second, a new step is introduced in which the captured and PCR-amplified target molecules are concatenated via incubation with the enzyme master mix for 1 hour. Notably, this adaptation differs from our multiplex PCR proof-of-concept demonstration in that the ConcatSeq adapters are ligated to the DNA fragments instead of being incorporated by PCR amplification.

To confirm that ConcatSeq can also be used with adapter-ligated DNA fragments, we generated a pool consisting of four PCR products from the human EGFR locus. The amplicons all had a size of 220 bp (Fig. 4b and Supplementary Table S3) and were amplified using male human genomic DNA (G1471, Promega) as a template. The pooled DNA was split into two aliquots, and two types of overlapping adapters were attached via A-tailed ligation. We performed a PCR step for enrichment prior to concatenation as described before in Fig. 1a. Note, that this PCR reaction mimics the PCR step in the SeqCap workflow in which the target-enriched library is amplified before sequencing. The two libraries were then mixed and concatenated as described before and then sequenced on a PacBio RSII sequencer. Average numbers of fragments per read were slightly reduced compared to previous runs (4.43 versus > 5 fragments per read; Table 1). However, on-target rate was again very high (96.9%) and the large majority of deconcatenated fragments had the expected size of 220 bp (Fig. 4c).

For a second test, we used a low molecular weight DNA ladder (LMW) containing 11 double-stranded DNA fragments of varying lengths present in non-equimolar concentrations as a starting material for adapter ligation. In this concatenation experiment, the average number of fragments per read was only 3.8-fold (Table 1), which is perhaps best explained by the presence of much larger molecules (up to 766 bp) in the mix. We noticed that representation of the LMW fragments was strongly influenced by adapter ligation and/or subsequent PCR amplification (Supplementary Fig. 2). A high correlation (Pearson’s r = 0.971) was found between the frequencies of the aligned LMW fragments and fragment concentrations after adapter ligation (Supplementary Fig. 2), validating that our method subsamples the molecules without introducing a large bias during assembly.

Taken together, these two experiments confirm that ConcatSeq can be readily adapted to workflows in which concatenation adapters are appended to the ends of target sequences by ligation as is the case in Roche Nimblegen’s SeqCap.

## Discussion

We describe a novel library preparation method, ConcatSeq, that is capable of increasing sequencing throughput of single molecule sequencing platforms. Fragment size appears to be an important factor that determines the degree of concatenation (and therefore the improvement in throughput) that can be achieved with this method. Short fragments (such as the amplicon from exon 3 of the NRAS locus) resulted in more than a 5-fold increase in throughput, while DNA samples that contained longer fragments (*e.g*. LMW ladder) yielded lower degrees of concatenation (~3.8 fold). This suggests that ConcatSeq is possibly best applied on circulating tumor DNA^20^ or DNA extracted from formalin-fixed paraffin-embedded tissues^21^ both of which are shorter than 200 bp in length.

Here, we calculated the improvement of throughput based on the average number of fragments detected across all sequencing reads. It is noteworthy, however, that much longer concatemers, consisting of up to 50 fragments, were detected in some of our experiments. Thus, ConcatSeq has the potential to increase the sequencing throughput beyond the five-fold that was observed here by applying size selection to the library before sequencing. For example, SPRIselect-based purification or preparative gel electrophoresis could be employed to more stringently remove shorter concatemers and enrich for longer molecules. It is important to bear in mind, however, that the accuracy of PacBio’s SMRT technology depends on circular consensus sequence (CCS) reads determined from multiple passes across both strands of the template^34^. Thus, there exists an inherent upper limit to the length of concatemers that yield useful sequencing information. Current statistics show PacBio’s accuracy reaches up to 99% with 5 complete passes^35^ and the average length of polymerase reads is between 10–15 kb^18^, making 2–3 kb the ideal length of a concatenated sequencing library. Assuming that short fragments generated by target enrichment workflows are typically around or smaller than 200 bp (*e.g*. ctDNA or DNA from FFPET), optimization of ConcatSeq should potentially be able to increase PacBio sequencing throughput to 10 and 15-fold. However, we note that such an approach might require a scale-up of input material to ensure the presence of enough long concatemers in the sample. Based on the data presented in Fig. 2e we estimate that ~7.9% of the reads contain 10 or more fragments, therefore requiring ~12.6 times more concatenated library to go into the size selection step. This seems feasible given that in the experiments presented here only a small fraction (~2.5–10%) of the library generated by ConcatSeq was used for a sequencing run on RSII.

Future strategies to optimize sequencing throughput would likely require not only a size selection to enrich for longer concatenated molecules, but also a means to control the maximum length of concatemers generated during the concatenation reaction. One strategy to limit the length of the molecules is to use spike-ins of adapters that are unable to participate in the concatenation reaction, such as PacBio-specific hairpin adapters, and in turn will effectively cap a molecule on one or both ends preventing it from growing further. The starting concentration of such ‘toxic’ adapters moreover could be used to control the size distribution of the final library.

We validated our approach by examining its feasibility for correctly detecting known SNVs in a well-characterized DNA sample. ConcatSeq correctly determined allele frequencies (Fig. 3b), and the representation of molecules in the concatenated samples was highly concordant with the original pool (Fig. 3d). This suggests that the method described here does not have a significant impact on error rate. The accuracy of our assay could be further improved by only including ‘high-quality’ reads, *e.g*. CCS reads with at least 5 passes, and/or balancing the PCR reactions to ensure equimolar representation of each amplicon. While we focused our attention on an oncology target panel with very short fragments (between 80–220 bp in length), the experiments using the LMW ladder (Supplementary Fig. 2) demonstrate that ConcatSeq can concatenate longer DNA fragments and is therefore applicable to other research areas.

Our method can be readily adapted to various target enrichment methods. We demonstrated this for multiplex PCR and workflows where sequencing adapters are incorporated through ligation (*e.g*. SeqCap). However, similar solutions can be applied to other workflows, such as HEAT-Seq, which was recently commercialized by Roche Nimblegen and is based on molecular inversion probes. In this case, the only modification to the original protocol is the use of primers that contain ConcatSeq adapters during the amplification of the circularized target molecule. The method described here can easily be adapted to different target enrichment schemes and minimally modifies their original workflows, adding only the cost for the Gibson Assembly master mix and the incubation time (1 hour in the current study) during concatenation. Thus, ConcatSeq is presented as a versatile new sample preparation tool for long-read sequencing technologies, including but not limited to PacBio platforms.

## Methods

### DNA, oligonucleotides, reagents, and kits

Genomic DNA was purchased from Horizon Discovery (HD701) and Promega (G1471). Low molecular weight DNA ladder was purchased from New England BioLabs (N3233). Oligonucleotides and Nuclease-Free Duplex Buffer were purchased from Integrated DNA Technologies. NEBuilder HiFi DNA Assembly Master Mix and Phusion High-Fidelity DNA Polymerase were purchased from New England BioLabs (E2621). Exonuclease III (M0379) and Exonuclease VII (M0206) were purchased from New England BioLabs. AmpliTaq Gold DNA Polymerase with Buffer II and MgCl_2_ (N8080241), Nuclease-Free Water (AM9937), and reagents for Qubit dsDNA assays (Q32850 and Q32851 for Broad Range [BR] and High Sensitivity [HS] Kit, respectively) were purchased from Thermo Fisher Scientific. KAPA Hyper Prep Kit (KK8503) and KAPA Pure Beads (KK8002) were purchased from KAPA BioSystems. Agilent DNA7500 kits (5067–1504) for the Agilent 2100 Bioanalyzer system were purchased from Agilent Technologies.

### PCR amplification and concatenation of target molecules

For experiments described in Figs 1 and 2, target regions of the genome were first amplified using gene-specific primers and 30 ng of HD701 of genomic DNA using AmpliTaq Gold DNA polymerase. This first round of PCR amplified the target regions together with flanking spacers on both ends of each amplicon (Supplementary Table S1 lists all primers used in this study). The resulting PCR product was then amplified with two primer pairs (Pr371/Pr372 and Pr373/Pr374) that prime off the spacer sequences and incorporate complementary ConcatSeq adapters to both ends in two separate PCR reactions. For experiments described in Fig. 3, the 20 target regions were first amplified in two separate PCR reactions due to primer incompatibilities. The first PCR mix amplified 11 targets: KRAS_Exon_2, KRAS_Exon_3, KRAS_Exon_4.1, NRAS_Exon_3, NRAS_Exon_4.1, NRAS_Exon_4.2, PIK3CA_Exon_9, BRAF_Exon_11, EGFR_Exon_18, EGFR_Exon_20.2, EGFR_Exon_21. The second PCR mix amplified 9 targets: KRAS_Exon_4.2, NRAS_Exon_2, PIK3CA_Exon_1, PIK3CA_Exon_4, PIK3CA_Exon_7, PIK3CA_Exon_20, BRAF_Exon_15, EGFR_Exon_19, EGFR_Exon_20.1 (Supplementary Table S3). The two resulting PCR products were subsequently amplified using primer pairs Pr371/Pr372 and Pr373/Pr374, respectively, in order to incorporate complementary ConcatSeq adapters to their ends. Resulting PCR products were then cleaned using KAPA Pure Beads at a 2x ratio by adding 100 μl of beads to 50 μl of PCR reaction and eluted into 50 μl of PCR-grade water. DNA concentration was quantified using the Qubit dsDNA BR Assay Kit. 200–300 ng of each of the two PCR products were then mixed and the final volume was brought to 40 μl with PCR-grade water. An equal volume (40 μl) of NEBuilder HiFi DNA Assembly Master Mix was added and incubated for 60 min at 50 °C. Gibson Assembly was followed by a clean-up step using KAPA Pure beads (0.8x ratio), followed by Qubit quantification (typically the concentration was ~10 ng/μl) and size range analysis using Agilent’s DNA7500 assay.

### Ligation of ConcatSeq adapters to target molecules prior to concatenation

For experiments described in Fig. 4, two different complementary T-tailed ConcatSeq adapters were generated by annealing Pr185 with Pr186 and Pr187 with Pr188 at 20 μM final concentration. Four different regions of the EGFR locus were amplified from human genomic DNA (male) from Promega using primers Pr001-Pr008 and Phusion High-Fidelity DNA Polymerase. The concentration of PCR products was determined using Qubit dsDNA BR Assay and then pooled at equimolar concentration (~73 nM). For the experiment described in Supplementary Fig. 2, LMW DNA ladder from New England BioLabs was diluted to 10 ng/μl and used as input material. The DNA samples were split into two reactions (with 25 μl comprising ~250 ng total DNA amount each) and subjected to the KAPA Hyper Prep assay: end-repair, A-tailing, and ligation to the two T-tailed ConcatSeq adapters. The resulting adapter-ligated fragment pools were then PCR-amplified using either primers Pr185 and Pr186 or Pr187 and Pr188, to enrich for the fragments that had successfully ligated adapters on both ends. DNA concentrations were quantified using the Qubit dsDNA BR Assay Kit. 200–300 ng of each of the two PCR products were then mixed and filled up to 40 μl with PCR-grade water. An equal volume of NEBuilder HiFi DNA Assembly Master Mix was added and incubated for 60 min at 50 °C. Gibson Assembly was followed by clean-up step using KAPA Pure beads (0.8x ratio), followed by Qubit quantification and size range analysis using Agilent’s DNA7500 assay.

### PacBio library preparation

Approximately 100 ng of the concatenated pool was used to prepare PacBio sequencing libraries using the KAPA Hyper Prep Kit. A suitable T-tailed hairpin adapter was first created by self-annealing of oligonucleotide Pr375 (20 μM) using Duplex Buffer and heating for 5 min to 80 °C followed by a slow ramp-down (0.2 °C per second) to 25 °C. Double-stranded DNA concatemers were then subjected to end-repair and A-tailing, and ligated to the hairpin adapters (at roughly a 250:1 ratio of adapters to concatemers) for 30 min at 20 °C. Unreacted T-tailed hairpin adapters and concatenated DNA molecules were removed by adding exonuclease III and exonuclease VII (1 μl of each) to the sample and incubating for 30 min at 37 °C. The resulting library molecules were cleaned-up with KAPA Pure Beads (0.8x ratio), and then quantified using Qubit dsDNA HS Assay. On average the final concentration of the sequencing libraries was between 0.5 and 2 ng/μl.

### PacBio sequencing

Binding Calculator (version 2.3.1) was used to prepare the library for PacBio sequencing using the MagBead one-cell per well (OCPW) protocol, and binding kit P6v2 was used with an on-plate concentration of 0.05 nM. Primer conditioning and annealing, as well as binding of the polymerase to the templates, and complex binding to the magnetic beads was done exactly as indicated by the binding calculator protocol. Template complexes were incubated with MagBeads for 2 hours at 4 °C prior to loading onto a SMRT cell. Four-hour movies were recorded and primary sequence analysis was performed on the PacBio RSII instrument.

### Secondary and Tertiary data analysis

Reads Of Inserts (ROI) were determined using the default settings on the SMRT Portal: only reads with more than one full pass and a minimum predicted accuracy of 90% were included for CCS read generation. The circular consensus sequence reads were deconcatenated using an adapter scanning approach, which we implemented in R. Briefly, a window of 30 bp (which corresponds to the length of the ConcatSeq adapter) slides along each read and performs approximate matching to the ConcatSeq adapter sequence (in forward and reverse complement orientation) using the *agrep* function and allowing for up to 4 mismatches, including insertions, and/or deletions. Adapters identified this way are removed from the reads leaving deconcatenated fragments behind. Supplementary Data S1 contains the R-script that was used for deconcatenation. In order to run the script follow these steps: (1) make sure that the *ShortRead* package^36^ for FASTQ input and manipulation has been installed from Bioconductor, (2) ‘copy and paste’ the entire F.DECONCAT function from Supplementary Data S1 into the R-console, and (3) type F.DECONCAT() and navigate to the fastq-file when prompted. We have noticed that fastq-files downloaded from NCBI’s SRA website sometimes have an empty line appended to their end. Remove this empty line before running F.DECONCAT().

New fastq files are created which list all adapters and fragments identified by this method. Before alignment of the fragments to the references, all fragments of length 1 bp were removed. Spacer sequences (introduced during the first PCR amplification in experiments described in Figs 1–3) remained part of each fragment after deconcatenation and were not specifically removed before alignment using bwa mem^37^. Spacer sequences flanking each fragment were soft-clipped during alignment. Only alignments that had a samflag of either 0 or 16, indicating a correct alignment in forward or reverse complement orientation, respectively, were kept for further analysis. In order to identify unintended hybrid fragments that were concatenated outside of the Gibson Assembly adapter region we inspected all alignments that had a samflag of either 256 or 272 (indicating multiple alignment in forward or reverse complement orientation, respectively). In the experiment presented in Fig. 2, a total of 633 of such cases were identified. In 224 of these a Gibson Assembly was identified using a more relaxed scanning approach (*i.e*. allowing for up to 6 mismatches including deletions and insertions). In the remaining 409 cases (corresponding to ~0.49% of all fragments identified in this sequencing run) two or more fragments had been concatenated outside of the adaptor region. For Fig. 3, we generated pileups of the aligned fragments using the *mpileup* function in samtools^38^. We used a Perl script to transform the pileups into contingency tables reporting the frequency of each base called at every position. Allele frequencies at the relevant positions (*i.e*. the known single nucleotide variants in HD701^31^) were extracted from these tables and plotted as the fraction of total number of reads aligned at that position (Supplementary Table S4).

## Electronic supplementary material

Supplementary Information
