# Supplementary material for: ConcatSeq: A method for increasing throughput of single molecule sequencing by concatenating short DNA fragments
Source: Sci Rep. 2017 Jul 12;7:5252. doi: 10.1038/s41598-017-05503-w (PMC5507877; doi:10.1038/s41598-017-05503-w)
Supplement: Supplementary file 1 — Supplementary Information [file 41598_2017_5503_MOESM1_ESM.pdf]

Supplementary Information

ConcatSeq: A method for increasing throughput of single molecule sequencing by concatenating short DNA fragments

Ulrich Schlecht[1]*, Janine Mok[1], Carolina Dallett[1], Jan Berka[1]

[1]Roche Sequencing Solutions, 4300 Hacienda Drive, Pleasanton, CA 94588

* Corresponding author
Email: ulrich.schlecht@roche.com

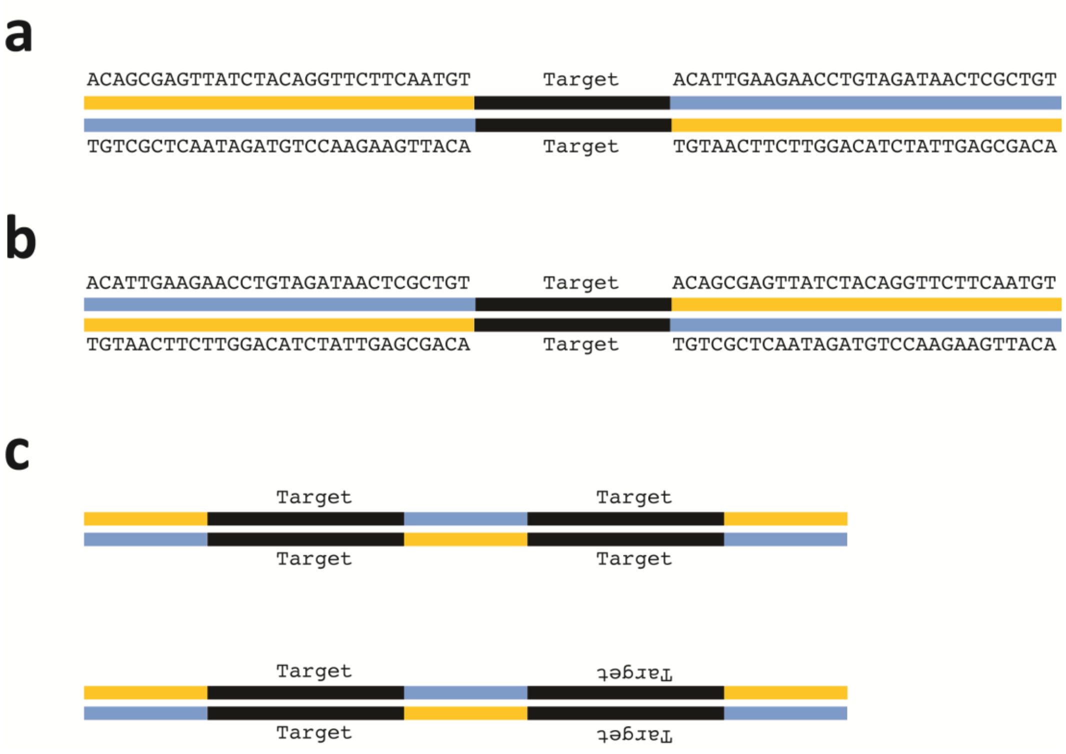

Supplementary Figure 1. Schematic of ConcatSeq adapters attached to target sequences and how these units assemble during concatenation. Each adapter consists of two strands (colored in yellow and blue). Portions depicted in blue are the reverse complement of those depicted in yellow and vice versa. Adapters are incorporated via PCR or A-tailed ligation. Strands colored in yellow (or blue) are identical when read in 5’ to 3’ orientation. Two types of adapters were designed for the current study: one in which the top strand and the bottom strand are of the yellow type in the adapters up- and down-stream of the target, respectively (a), and one in which the bottom strand and the top strand are of the yellow type in the adapters up- an down-stream of the target, respectively (b). The ‘concatenation units’ shown in (a) and (b) can assemble in two different ways as depicted in (c): one in which the targets on the top (*i.e.* the sequenced) strand are in the same orientation relative to each other, and one in which the targets are in opposite orientation (*i.e.* forward and reverse complement) to each other.

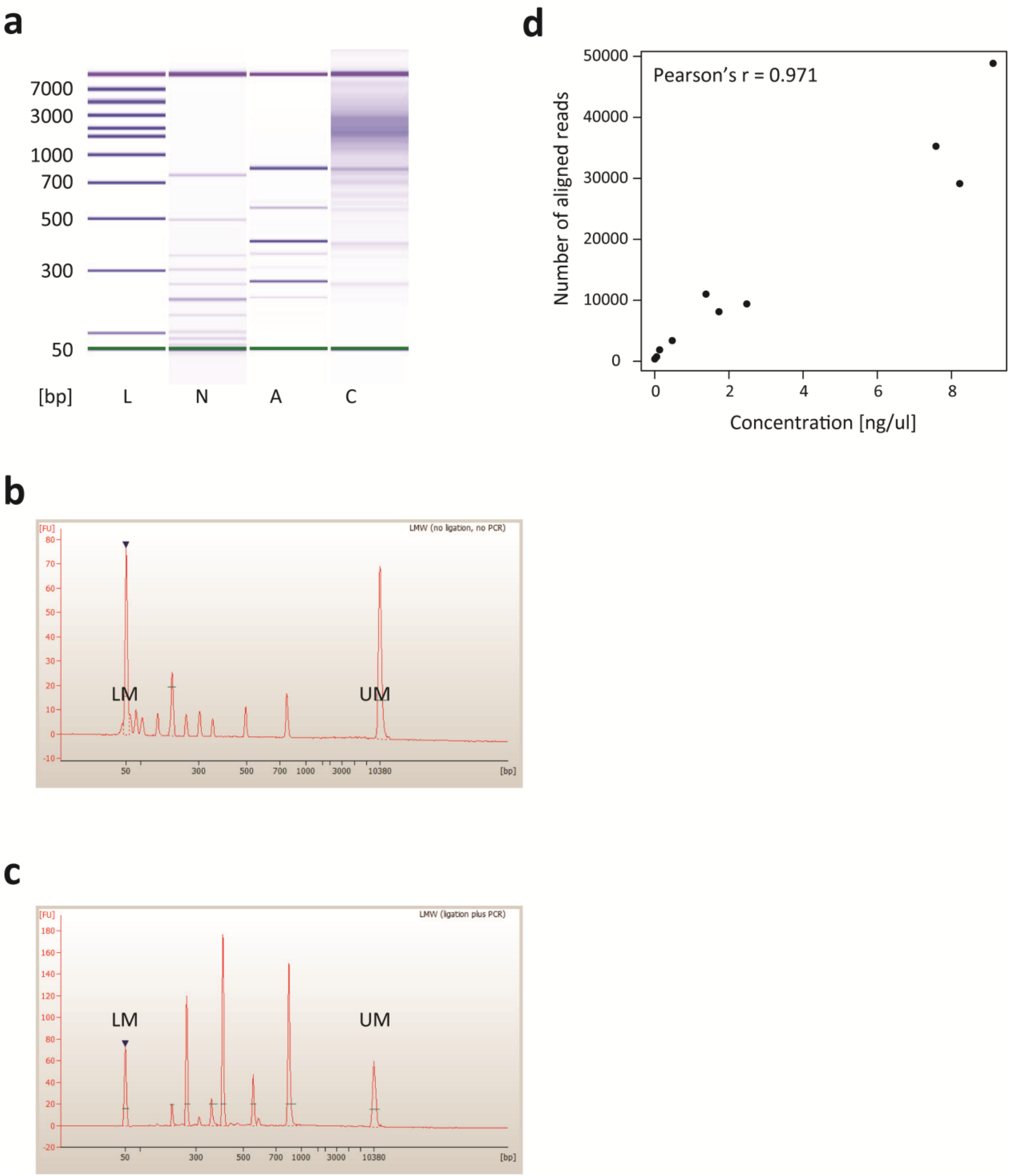

Supplementary Figure 2. ConcatSeq using low molecular weight DNA ladder as input material. (a) Bioanalyzer DNA7500 gel image showing ladder [L], the non-concatenated pool of fragments present in the low molecular weight DNA ladder (LMW) [N], the non-concatenated DNA pool with adapters ligated [A], and the concatenated sample [C]. (b) Bioanalyzer DNA7500 electropherogram of 10 ng of unmodified LMW. The lower marker (LM) runs at 50 bp. The upper marker (UM) runs at 10,380 bp. (c) Bioanalyzer DNA7500 electropherogram of an aliquot of LMW DNA ladder after ligation to ConcatSeq adapters and PCR amplification using primers that amplify only the fragments that have ConcatSeq adapters present on both ends. (d) Scatterplot comparing number of aligned fragments after deconcatenation of reads from LMW-concatemer sequencing run and concentration of fragments detected in the adapter-ligated and PCR-amplified LMW (see panel c).

Supplementary Table S1. Primers used in this study.

Primer Name	Primer Sequence
F_NRAS_E3NX	TCGTCGGCAGCGTCAGATGTGTATAAGAGACAGTCTACAAAGTGGTTCTGGATTAG
R_NRAS_E3NX	GTCTCGTGGGCTCGGAGATGTGTATAAGAGACAGCTGGTTTCCAACAGGTTCTT
F_NRAS_E4.1NX	TCGTCGGCAGCGTCAGATGTGTATAAGAGACAGTTTAGGGAGCAGATTAAGCGAGTA
R_NRAS_E4.1NX	GTCTCGTGGGCTCGGAGATGTGTATAAGAGACAGGGCTTGTTTTGTATCAACTGTCCTTGTT
F_NRAS_E4.2NX	TCGTCGGCAGCGTCAGATGTGTATAAGAGACAGACTGGCCAAGAGTTACGGGATT
R_NRAS_E4.2NX	GTCTCGTGGGCTCGGAGATGTGTATAAGAGACAGTCTACCAGAGTTAATCAACTGATGCA
F_PIK3CA_E9NX	TCGTCGGCAGCGTCAGATGTGTATAAGAGACAGGACAAAGAACAGCTCAAAGCAATTTCTA
R_PIK3CA_E9NX	GTCTCGTGGGCTCGGAGATGTGTATAAGAGACAGATCTCCATTTTAGCACTTACCTGTGACT
F_EGFR_E18NX	TCGTCGGCAGCGTCAGATGTGTATAAGAGACAGAGCTGTCTTGAGGATCTTGAAGGAA
R_EGFR_E18NX	GTCTCGTGGGCTCGGAGATGTGTATAAGAGACAGCCTGTGCCAGGGACCT
F_EGFR_E20.2NX	TCGTCGGCAGCGTCAGATGTGTATAAGAGACAGGGGCATCTGCCTCACCTCCA
R_EGFR_E20.2NX	GTCTCGTGGGCTCGGAGATGTGTATAAGAGACAGATTGTCTTTGTGTTCCCGGACATA
F_EGFR_E21NX	TCGTCGGCAGCGTCAGATGTGTATAAGAGACAGGTGAAAACACCGCAGCATGTCAA
R_EGFR_E21NX	GTCTCGTGGGCTCGGAGATGTGTATAAGAGACAGCAGGAAAATGCTGGCTGACCTAA
F_BRAF_E11NX	TCGTCGGCAGCGTCAGATGTGTATAAGAGACAGGTCACAATGTCACCACATTACATAC
R_BRAF_E11NX	GTCTCGTGGGCTCGGAGATGTGTATAAGAGACAGAGATTCCTGATGGGCAGATTAC
F_KRAS_E2NX	TCGTCGGCAGCGTCAGATGTGTATAAGAGACAGTATCGTCAAGGCACTCT
R_KRAS_E2NX	GTCTCGTGGGCTCGGAGATGTGTATAAGAGACAGATTATAAGGCCTGCTGAAA
F_KRAS_E3NX	TCGTCGGCAGCGTCAGATGTGTATAAGAGACAGCATGTACTGGTCCCTCATTG
R_KRAS_E3NX	GTCTCGTGGGCTCGGAGATGTGTATAAGAGACAGTCCCTTCTCAGGATTCCTAC
F_KRAS_E4.1NX2	TCGTCGGCAGCGTCAGATGTGTATAAGAGACAGTGTGTCTACTGTTCTAGAAGGC
R_KRAS_E4.1NX2	GTCTCGTGGGCTCGGAGATGTGTATAAGAGACAGGGACTCTGAAGATGTACCTATGG
F_NRAS_E2_NX	TCGTCGGCAGCGTCAGATGTGTATAAGAGACAGTTCGCCTGTCCTCATGTATT
R_NRAS_E2_NX	GTCTCGTGGGCTCGGAGATGTGTATAAGAGACAGTGGTGAAACCTGTTTGTTGG
F_PIK3CA_E1NX	TCGTCGGCAGCGTCAGATGTGTATAAGAGACAGGTTACTCAAGAAGCAGAAAGGGAAGA
R_PIK3CA_E1NX	GTCTCGTGGGCTCGGAGATGTGTATAAGAGACAGCACGGTTGCCTACTGGTTCAATTACT
F_PIK3CA_E4NX	TCGTCGGCAGCGTCAGATGTGTATAAGAGACAGCCTTTGGGTTATAAATAGTGCACTCAGA
R_PIK3CA_E4NX	GTCTCGTGGGCTCGGAGATGTGTATAAGAGACAGAGCATCAGCATTTGACTTTACCTTATCA
F_PIK3CA_E7NX	TCGTCGGCAGCGTCAGATGTGTATAAGAGACAGGTGTTTTGAAATGTGTTTTATAATTTAGACTAGTG
R_PIK3CA_E7NX	GTCTCGTGGGCTCGGAGATGTGTATAAGAGACAGCTAGAGTGTCTGTGTAATCAAACAAGTTTATATT
F_PIK3CA_E20NX	TCGTCGGCAGCGTCAGATGTGTATAAGAGACAGCTAGCCTTAGATAAAACTGAGCAAGAG
R_PIK3CA_E20NX	GTCTCGTGGGCTCGGAGATGTGTATAAGAGACAGTTAATTGTGTGGAAGATCCAATCCAT
F_EGFR_E19NX	TCGTCGGCAGCGTCAGATGTGTATAAGAGACAGGGATCCCAGAAGGTGAGAAAGTTAA
R_EGFR_E19NX	GTCTCGTGGGCTCGGAGATGTGTATAAGAGACAGCCCCACACAGCAAAGCAGAA
F_EGFR_E20.1NX	TCGTCGGCAGCGTCAGATGTGTATAAGAGACAGGCCTCTCCCTCCCTCCAGGAA
R_EGFR_E20.1NX	GTCTCGTGGGCTCGGAGATGTGTATAAGAGACAGCACGGTGGAGGTGAGGCA
F_BRAF_E15NX	TCGTCGGCAGCGTCAGATGTGTATAAGAGACAGGCCTCAATTCTTACCATCCA
R_BRAF_E15NX	GTCTCGTGGGCTCGGAGATGTGTATAAGAGACAGTTCATGAAGACCTCACAGTAAA
F_KRAS_E4.2NX	TCGTCGGCAGCGTCAGATGTGTATAAGAGACAGTCAGTGTTACTTACCTGTCTTGT
R_KRAS_E4.2NX	GTCTCGTGGGCTCGGAGATGTGTATAAGAGACAGCAGGCTCAGGACTTAGCAA
Pr371	ACAGCGAGTTATCTACAGGTTCTTCAATGTTCGTCGGCAGCGTC
Pr372	ACAGCGAGTTATCTACAGGTTCTTCAATGTGTCTCGTGGGCTCGG
Pr373	ACATTGAAGAACCTGTAGATAACTCGCTGTTCGTCGGCAGCGTC
Pr374	ACATTGAAGAACCTGTAGATAACTCGCTGTGTCTCGTGGGCTCGG
Pr185	/5Phos/ACAGCGAGTTATCTACAGGTTCTTCAATGT
Pr186	/5Phos/CATTGAAGAACCTGTAGATAACTCGCTGT
Pr187	/5Phos/CAGCGAGTTATCTACAGGTTCTTCAATGT
Pr188	/5Phos/ACATTGAAGAACCTGTAGATAACTCGCTGT
Pr001	TACGACGAGACGTCGCTCACTTACTGCTCATTATCACAGCGGTCAAAGGCTAAC
Pr002	TCTGACGGTGCAACCTGTGGTCAAGGTCTAAAATGGGTCCAGGGAGCC
Pr003	TCACGACGCGACGTCGTTCATATCATAATCCATAAATTATGATAGGTGGGACAGTGCACC

Pr004	TCTGGTGGAGCAACCTGTGCGCGCAGCACGGTGTTGACTC
Pr005	TGCTGCTGAGTGGTCTCGCTGTGCCCCAACCAAACGACCG
Pr006	TCTGGCTGGTGTCGTCACGTGTACGCCTTCTCTACAGAGCTTGACAG
Pr007	TCACGACGTGACGTCGTCCACCAGTTAACGTCTTCCTTCTCTCTCTGTCATAGGGAC
Pr008	TGCTGGTGGTGCAACCTGTGCCTGAGGTTCAGAGCCATGGACC
Pr375	ATCTCTCTCTTTTCCTCCTCCTCCGTTGTTGTTGTTGAGAGAGATT

Supplementary Table S2. Steps involved in generating ConcatSeq libraries.

Step #	Description
1	Perform a first round of PCR that amplifies target region(s) of interest with primers that contain gene-specific portion and flanking spacer sequences (*e.g.* primers F_NRAS_E3NX & R_NRAS_E3NX for amplifying part of the NRAS exon 3 locus).
2	The first round of PCR is followed by a second round of PCR which is performed in two separate reactions (see 2a and 2b).
2a	Perform a second round of PCR using an aliquot from the first PCR as template and primers 371 and 372 to incorporate Gibson Assembly adapters (50 ul reaction).
2b	Perform a second round of PCR using an aliquot from the first PCR as template and primers 373 and 374 to incorporate Gibson Assembly adapters (50 ul reaction).
3	Clean up the PCR product with KAPA Pure beads at a 2x ratio and elute the DNA into 50 ul of PCR-grade water.
4	Quantify DNA concentration using Qubit dsDNA BR Assay kit.
5	Mix 200 - 300 ng of both amplicons from the second round of PCR and bring the volume up to 40 ul using PCR-grade water.
6	Add 40 ul of NEBuilder HiFi DNA Assembly Master Mix to the sample.
7	Incubate reaction for 60 min at 50°C.
8	Clean up the concatenation product with KAPA Pure beads at a 0.8x ratio and elute the DNA into 20 ul of PCR-grade water.
9	Quantify DNA concentration using Qubit dsDNA BR Assay kit.
10	Perform size range analysis using Agilent's DNA7500 assay.
11	Use 100 ng of concatenated DNA to prepare PacBio sequencing library using the KAPA HyperPrep Kit.
12	Bring volume of sample up to 50 ul with PCR-grade water.
13	Perform End Repair and A-tailing with KAPA End Repair & A-tailing Buffer (7 ul) and KAPA End Repair and A-tailing Enzyme Mix (3 ul).
14	Incubate reaction for 30 min at 20°C and 30 min at 65°C.
15	Add 10 ul of PacBio SMRTbells (hairpin adapter) at a 250:1 ratio of (adapter:concatemer), KAPA Ligation Buffer (30 ul) and KAPA DNA Liagse (10 ul).
16	Incubate reaction for 30 min at 20°C.
17	Add 1 ul of exonuclease III and 1 ul of exonuclease VII.
18	Incubate reaction for 30 min at 37°C.
19	Clean up the sample with KAPA Pure beads at a 0.8x ratio and elute the DNA into 10 ul of PCR-grade water.
20	Quantify DNA concentration using Qubit dsDNA HS Assay kit.
21	The final concentration of the sequencing library should be between 0.5 and 2 ng/ul.

Supplementary Table S3. Genomic targets amplified in this study. Top table indicates names of primers that were used to generate target amplicon, chromosome, start and end position of the target sequence in reference genome GRCh37/hg19. Bottom table shows the sequence of the target sequences.

Amplicon Name	Forward primer	Reverse primer	Chromosome	Start position	End position
EGFR_Exon_18	F_EGFR_E18NX	R_EGFR_E18NX	chr7	55241655	55241755
EGFR_Exon_20.2	F_EGFR_E20.2NX	R_EGFR_E20.2NX	chr7	55249036	55249126
EGFR_Exon_21	F_EGFR_E21NX	R_EGFR_E21NX	chr7	55259475	55259604
KRAS_Exon_2	F_KRAS_E2NX	R_KRAS_E2NX	chr12	25398256	25398337
KRAS_Exon_3	F_KRAS_E3NX	R_KRAS_E3NX	chr12	25380242	25380357
KRAS_Exon_4.1	F_KRAS_E4.1NX2	R_KRAS_E4.1NX2	chr12	25378617	25378686
BRAF_Exon_11	F_BRAF_E11NX	R_BRAF_E11NX	chr7	140481347	140481456
NRAS_Exon_2	F_NRAS_E2_NX	R_NRAS_E2_NX	chr1	115256483	115256570
NRAS_Exon_3	F_NRAS_E3NX	R_NRAS_E3NX	chr1	115258693	115258812
NRAS_Exon_4.1	F_NRAS_E4.1NX	R_NRAS_E4.1NX	chr1	115252250	115252354
NRAS_Exon_4.2	F_NRAS_E4.2NX	R_NRAS_E4.2NX	chr1	115252130	115252244
PIK3CA_Exon_9	F_PIK3CA_E9NX	R_PIK3CA_E9NX	chr3	178936037	178936142
EGFR_Exon_19	F_EGFR_E19NX	R_EGFR_E19NX	chr7	55242422	55242539
EGFR_Exon_20.1	F_EGFR_E20.1NX	R_EGFR_E20.1NX	chr7	55248968	55249060
KRAS_Exon_4.2	F_KRAS_E4.2NX	R_KRAS_E4.2NX	chr12	25378534	25378613
BRAF_Exon_15	F_BRAF_E15NX	R_BRAF_E15NX	chr7	140453061	140453184
PIK3CA_Exon_1	F_PIK3CA_E1NX	R_PIK3CA_E1NX	chr3	178916830	178916938
PIK3CA_Exon_4	F_PIK3CA_E4NX	R_PIK3CA_E4NX	chr3	178921496	178921598
PIK3CA_Exon_7	F_PIK3CA_E7NX	R_PIK3CA_E7NX	chr3	178927916	178928031
PIK3CA_Exon_20	F_PIK3CA_E20NX	R_PIK3CA_E20NX	chr3	178952021	178952133
EGFR.Frag1	Pr001	Pr002	chr7	55210766	55210945
EGFR.Frag2	Pr003	Pr004	chr7	55221459	55221638
EGFR.Frag3	Pr005	Pr006	chr7	55241281	55241460
EGFR.Frag4	Pr007	Pr008	chr7	55242382	55242561

Amplicon Name	Amplicon Sequence
EGFR_Exon_18	agctctcttgaggatcttgaaggaaactgaattcaaaaagatcaaagtgctgggctccggtgcgttcggcacggtgtataaggtaaggtccctggcacagg
EGFR_Exon_20.2	gggcatctgcctcacctccaccgtgcagctcatcacgcagctcatgcccttcggctgcctcctggactatgtccgggaacacaaagacaat
EGFR_Exon_21	gtgaaaacaccgcagcatgtcaagatcacagattttgggctggccaaactgctgggtgcggaagagaaagaataccatgcagaaggaggcaaagtaaggaggtggctttaggtcagccagcattttcctg
KRAS_Exon_2	tatcgtcaaggcactcttgcctacgccaccagctccaactaccacaagtttatattcagtcattttcagcaggccttataat
KRAS_Exon_3	catgtactggtccctcattgcactgtactcctcttgacctgctgtgtcgagaatatccaagagacaggtttctccatcaattactacttgcttcctgtaggaatcctgagaaggga
KRAS_Exon_4.1	tgtgtctactgttctagaaggcaaatcacatttatttcctactaggaccataggtacatcttcagagtcc
BRAF_Exon_11	gtcacaatgtcaccacattacatacttaccatgccactttcccttgtagactgttccaaatgatccagatccaattctttgtcccactgtaatctgcccatcaggaatct
NRAS_Exon_2	ttcgcctgtcctcatgtattggtctctcatggcactgtactcttcttgtccagctgtatccagtatgtccaacaaacaggtttcacca
NRAS_Exon_3	tctacaaagtggttctggattagctggattgtcagtgcgcttttcccaacaccacctgctccaaccaccaccagtttgtactcagtcatttcacaccagcaagaacctgttggaaaccag
NRAS_Exon_4.1	ggcttgttttgtatcaactgtccttgttggcaaatcacacttgtttcccactagcaccataggtacatcatccgagtcttttactcgcttaatctgctccctaaa
NRAS_Exon_4.2	tctaccagagttaatcaactgatgcaaactcttgcacaaatgctgaaagctgtaccatacctgtctggtcttggctgaggtttcaatgaatggaatcccgtaactcttggccagt
PIK3CA_Exon_9	gacaaagaacagctcaaagcaatttctacacgagatcctctctctgaaatcactgagcaggagaaagattttctatggagtcacaggtaagtgctaaaatggagat
EGFR_Exon_19	ggatcccagaaggtgagaaagttaaaattcccgtcgctatcaaggaattaagagaagcaacatctccgaaagccaacaaggaaatcctcgatgtgagtttctgctttgctgtgtgggg

EGFR_Exon_20.1	gcctctccctccctccaggaagcctacgtgatggccagcgtggacaacccccacgtgtgccgcctgctgggcatctgcctcacctccaccgtg
KRAS_Exon_4.2	tcagtgttacttacctgtcttgtctttgctgatgtttcaataaaaggaattccataacttcttgctaagtcctgagcctg
BRAF_Exon_15	gcctcaattcttaccatccacaaaatggatccagacaactgttcaaactgatgggacccactccatcgagatttcactgtagctagaccaaaatcacctatttttactgtgaggtcttcatgaa
PIK3CA_Exon_1	gttactcaagaagcagaaagggaagaatttttgatgaaacaagacgactttgtgaccttcggctttttcaacccttttaaaagtaattgaaccagtaggcaaccgtg
PIK3CA_Exon_4	cctttgggttataaatagtgcactcagaataaaaattctttgtgcaacctacgtgaatgtaaatattcgagacattgataaggtaaagtcaaatgctgatgct
PIK3CA_Exon_7	gtgttttgaaatgtgttttataatttagactagtgaatatttttctttgtttttaaggaacactgtccattggcatggggaaatataaacttgtttgattacacagacactctag
PIK3CA_Exon_20	ctagccttagataaaactgagcaagaggctttggagtatttcatgaaacaaatgaatgatgcacatcatggtggctggacaacaaaaatggattggatcttccacacaattaa
EGFR.Frag1	ttactgctcattatcacaggggtcaaaggctaacgtgcagggattgttgcagatcgtggacatgctgcctcctgtgtccatgactgcaatcgtctacctattttacagttgttgagcactcgtgtgcattagggttcaactgggcgtcctagggctccctggacccattttagaccttga
EGFR.Frag2	tatcataatccataaattatgataggtgggacagtgcacctaagaaaaaaatggactttttagagaagggtctttctgactctgcagagggcgccagctgggttttcccacactagtggaacactaggctgcaaagacagtaacttgggctttctgacgggagtcaacaccgtgctgcgc
EGFR.Frag3	gtgccccaaccaaacgaccgccatgcacaacttccctaccggagttttcaatccagttaataggcgtggaaacagacatagaaattgtgtttgttgaaaggtagctgttcagttaaagaacacctgtatcagagcctgtgtttctaccaacttctgtcaagctctgtagagaaggcgtac
EGFR.Frag4	ccagttaacgtcttccttctctctctgtcatagggactctggatcccagaaggtgagaaagttaaaattcccgtcgctatcaaggaattaagagaagcaacatctccgaaagccaacaaggaaatcctcgatgtgagtttctgctttgctgtgtgggggtccatggctctgaacctcagg

Supplementary Table S4. Genomic positions and allele frequencies of verified mutations in HD701 (Quantitative Multiplex Reference Standard from Horizon Discovery), and frequencies determined in non-concatenated and concatenated samples (in triplicate).

Gene	Chromosome	Position (GRCh37/hg19)	Variant (amino acid)	Variant (base)	Position in amplicon in Supplementary Table S3	Expected Allele Frequency [%]	Observed Allele Frequency in non-concat [%]	Observed Allele Frequency in concat rep 1 [%]	Observed Allele Frequency in concat rep 2 [%]	Observed Allele Frequency in concat rep 3 [%]
EGFR	chr7	55241707	G719S	2155 G>A	53	24.5	30.49	30.21	29.84	30.64
EGFR	chr7	55242465	ΔE746 - A750	2235 G>del	44	2	3.38	4.15	3.78	3.84
EGFR	chr7	55242466	ΔE746 - A750	2236 G>del	45	2	3.23	2.99	2.96	2.84
EGFR	chr7	55242467	ΔE746 - A750	2237 A>del	46	2	3.36	3.42	3.32	3.05
EGFR	chr7	55242468	ΔE746 - A750	2238 A>del	47	2	3.5	2.96	2.99	2.81
EGFR	chr7	55242469	ΔE746 - A750	2239 T>del	48	2	3.77	3.54	3.49	3.43
EGFR	chr7	55242470	ΔE746 - A750	2240 T>del	49	2	3.09	3.05	2.88	2.69
EGFR	chr7	55242471	ΔE746 - A750	2241 A>del	50	2	3.09	4.07	4.09	3.75
EGFR	chr7	55242472	ΔE746 - A750	2242 A>del	51	2	2.96	2.82	2.69	2.62
EGFR	chr7	55242473	ΔE746 - A750	2243 G>del	52	2	3.23	2.84	2.75	2.75
EGFR	chr7	55242474	ΔE746 - A750	2244 A>del	53	2	3.09	2.76	2.74	2.48
EGFR	chr7	55242475	ΔE746 - A750	2245 G>del	54	2	3.09	2.84	2.82	2.48
EGFR	chr7	55242476	ΔE746 - A750	2246 A>del	55	2	3.23	3.15	3.19	2.95
EGFR	chr7	55242477	ΔE746 - A750	2247 A>del	56	2	3.23	2.67	2.66	2.48
EGFR	chr7	55242478	ΔE746 - A750	2248 G>del	57	2	3.36	2.87	2.82	2.66
EGFR	chr7	55242479	ΔE746 - A750	2249 C>del	58	2	3.49	2.77	2.78	2.65
EGFR	chr7	55249071	T790M	2369 C>T	36	1	1.23	1.33	0.86	0.95
EGFR	chr7	55259515	L858R	2573 T>G	41	3	2.01	2.5	2.3	1.92
BRAF	chr7	140453136	V600E	1799 T>A	76	10.5	5.07	8.35	8.12	8.65
KRAS	chr12	25398281	G13D	38 G>A	26	15	17.49	15.6	15.57	14.89
KRAS	chr12	25398284	G12D	35 G>A	29	6	9	9.34	8.29	9.22
NRAS	chr1	115256530	Q61K	181 C>A	48	12.5	6.27	8	8.26	7.63
PIK3CA	chr3	178936091	E545K	1633 G>A	55	9	7.84	8.15	7.45	7.43
PIK3CA	chr3	178952085	H1047R	3140 A>G	65	17.5	14.49	16.99	16.46	16.13

Supplementary Data S1. The R-script used for deconcatenation. Comments are colored in green.

```
F.DECONCAT <- function() {

	# make sure that ShortRead package has been loaded
	require(ShortRead)

	# ask user to navigate to fastq-file
	fileName=file.choose()

	# clip off file extension
	fileStem <- sub(".fastq", "", fileName)

	# in some cases the last line has to be removed from the fastq-file
	fastq <- readFastq(fileName)

	# extract the headers
	iden <- as.vector(id(fastq))

	# extract the reads
	read <- as.vector(sread(fastq))

	# extract the quality scores
	qual <- as.vector(quality(quality(fastq)))

	# create a function for splitting vectors
	splitAt <- function(x, y) {
		out <- list()
		pos <- c(1, y+1, length(x)+1)
		for (i in seq_along(pos[-1])) {
			out[[i]] <- x[pos[i]:(pos[i+1]-1)]
		}
		return(out)
	}

	# these are the objects that'll hold information about the insert sequences
	insSeqn <- c()
	insQual <- c()
	insHead <- c()
	insInfo <- c()

	# these are the objects that'll hold information about the adapter sequences
	adaSeqn <- c()
	adaQual <- c()
	adaHead <- c()
	adaInfo <- c()

	# cycle through the reads
	for (j in 1:length(read)) {
		# indicate to user that the script is running and how far it has progressed
		if (j %% 20 == 0) { print(paste(j, "of", length(read), "reads have been analyzed so far", sep=" ")) }

		# some preparation: get current read, header, quality scores et cetera
		idn <- iden[j]		# get identifier
		qua <- qual[j]		# get quality scores
		seq <- read[j]		# get read sequence
		dis <- c()		# match within the defined distance?
		std <- c()		# match to which strand?
		wdw <- c()		# sequence inside the sliding window
		fwd <- "ACAGCGAGTTATCTACAGGTTCTTCAATGT"
		rvr <- "ACATTGAAGAACCTGTAGATAACTCGCTGT"
		ada <- c(fwd, rvr)	# lookup vector for forward and reverse adapter sequence
		numMis <- 4		# number of allowed mismatches

		# create a sliding window of 30 bp and calculate distance to adapter sequence
		for (i in 1:(nchar(seq)-29)) {
			pat <- substring(seq, i, i+29)			# get the sequence inside the sliding window
			d1 <- length(agrep(fwd, pat, numMis))		# do approximate grep on forward adapter sequence
			d2 <- length(agrep(rvr, pat, numMis))		# do approximate grep on revcom adapter sequence
			if (d1 == 1) { dis <- c(dis, d1); std <- c(std, 1); wdw <- c(wdw, pat); }
			if (d1 == 0) { dis <- c(dis, d2); std <- c(std, 2); wdw <- c(wdw, pat); }
		}

		# extract position of adapters
		pos <- which(dis==1)	# these are the positions where there is a match within the edit distance
		dif <- diff(pos)	# identify consecutive positions that match within the edit distance
		spl <- which(dif > 1)	# here is where a new match region begins
		spl <- splitAt(pos, spl) # the match regions separated into individual vectors
```

```
		# loc will hold the position at the center of each region and therefore presumably the best match
		loc <- c()
		for (i in 1:length(spl)) {
			loc <- c(loc, spl[[i]][length(spl[[i]])%/%2 + 1])
		}

		# sta and end will contain the start and end position of the adapter sequence
		sta <- c()
		end <- c()
		for (k in 1:length(loc)) {
			adapSeq <- ada[std[loc[k]]]
			readSeq <- substring(seq, loc[k], loc[k]+29)
			ads <- adist(adapSeq, readSeq, partial=TRUE, counts=T)
			off <- as.vector(attr(ads, "offsets"))
			sta <- c(sta, loc[k]+off[1]-1)
			end <- c(end, loc[k]+off[2]-1)
		}

		# loop through adapter coordinates
		for (i in 1:length(sta)) {
			adaSeqn <- c(adaSeqn, substring(seq, sta[i], end[i]))
			adaQual <- c(adaQual, substring(qua, sta[i], end[i]))
			adaHead <- c(adaHead, paste(idn, sta[i], end[i], sep="-"))
		}
		adaInfo <- c(adaInfo, idn, nchar(seq), length(sta))

		# jump to next read if there is no match to an adapter at all
		if (length(loc)==0) { next; }

		# create a lookup table for start and end coordinates of inserts
		mat <- matrix(sort(c(1, sta-1, end+1, nchar(seq))), ncol=2, byrow=T)

		# number of inserts
		num <- (dim(mat)[1])

		# loop through coordinates to retrieve sequences
		for (i in 1:num) {
			insSeqn <- c(insSeqn, substring(seq, mat[i,1], mat[i,2]))
			insQual <- c(insQual, substring(qua, mat[i,1], mat[i,2]))
			insHead <- c(insHead, paste(idn, mat[i,1], mat[i,2], sep="-"))
		}
		insInfo <- c(insInfo, idn, nchar(seq), num)
	}

	# write new files that contain information about inserts and adapters
	write.table(file=paste(fileStem, "_inserts.txt", sep=""), cbind(insHead, nchar(insSeqn), insSeqn), col.names=F, row.names=F, quote=F, sep="\t")
	write.table(file=paste(fileStem, "_adapter.txt", sep=""), cbind(adaHead, nchar(adaSeqn), adaSeqn), col.names=F, row.names=F, quote=F, sep="\t")

	# create new fastq file with deconcatenated sequences (inserts)
	mat <- cbind(insHead, insSeqn, "+", insQual)
	newFastq <- as.vector(unlist(t(mat)))
	write.table(newFastq, file=paste(fileStem, "_inserts_decon.fastq", sep=""), col.names=F, row.names=F, quote=F)

	# create new fastq file with deconcatenated sequences (adapters)
	mat <- cbind(adaHead, adaSeqn, "+", adaQual)
	newFastq <- as.vector(unlist(t(mat)))
	write.table(newFastq, file=paste(fileStem, "_adapter_decon.fastq", sep=""), col.names=F, row.names=F, quote=F)

	# create file that summarizes information about read length and number of fragments in each read
	write.table(matrix(insInfo, ncol=3, byrow=T), file=paste(fileStem, "_inserts-Info.txt", sep=""), col.names=F, row.names=F, quote=F, sep="\t")
}
```
